# Initially Suspected Anaphylaxis Following Iodinated Contrast: Jod-Basedow Phenomenon in a 73-Year-Old Female Without a History of Thyroid Dysfunction

**DOI:** 10.7759/cureus.38415

**Published:** 2023-05-01

**Authors:** Devina Adalja, Brooke E Kania, Isaac M Soliman, Jessimar Sanchez, Haris Kalatoudis

**Affiliations:** 1 Internal Medicine, St. Joseph’s Medical Center, Paterson, USA; 2 Medicine, Rowan School of Osteopathic Medicine, Stratford, USA; 3 Critical Care Medicine, St. Joseph’s Medical Center, Paterson, USA

**Keywords:** jod-basedow effect, critical care, imaging contrast, hyperthyroidism, iodine-induced thyrotoxicosis

## Abstract

The Jod-Basedow phenomenon (JB phenomenon), also referred to as “iodine-induced hyperthyroidism,” rarely occurs. Radiological imaging using iodinated contrast contains a dose of 300 to 1221 mg of iodine per kilogram, which can transiently induce clinically significant hyperthyroidism (referred to as Jod-Basedow Syndrome) in euthyroid patients. Hence, the reporting of such events is important for clinicians to be aware of, to prevent unnecessary iodine-based imaging. Underlying thyroid abnormalities, including latent Graves’ disease, autoimmune thyroiditis, use of iodine-containing foods or medications, such as amiodarone, and Lugol’s iodine have been shown to increase the risk of JB phenomenon. In terms of the pathophysiology of the JB phenomenon, when iodine exposure is in excess, increased iodine leads to increased hormone synthesis, and with an absence of auto-regulation, this can lead to thyrotoxicosis. In this case report, we describe the iodine-induced JB phenomenon in a 73-year-old female with no prior thyroid dysfunction, who was initially admitted for pyelonephritis and was eventually transferred to the intensive care unit secondary to suspected anaphylaxis.

## Introduction

Iodine-induced hyperthyroidism (IHT) or the Jod-Basedow phenomenon (JB phenomenon) represents a rare and relatively underreported event. This manifests typically in patients who undergo radiological imaging using iodinated contrast [1]. This phenomenon is more commonly experienced in patients with pre-existing Graves’ disease, multinodular goiter, or thyrotoxicosis [2]. In a study evaluating a geriatric population of patients, 15 out of 73 patients without underlying thyroid conditions had increased levels of free serum thyroxine (t4) following contrast-based imaging, where two of these patients experienced symptoms related to thyroid dysfunction [3]. Therefore, it is important to recognize the JB phenomenon and understand the clinical significance behind the disease process. In this case report, we describe the occurrence of the JB phenomenon in a 73-year-old female with no prior history of thyroid dysfunction, who was upgraded to the intensive care unit (ICU) for suspected anaphylaxis.

## Case presentation

A 73-year-old Middle Eastern female with a past medical history of hypertension, dyslipidemia, tobacco use disorder, lung cancer status post two lung resections (first resection 30 years prior, second resection one year prior), and a right hip total arthroplasty one month prior, presented to the emergency department (ED) with the chief complaint of abdominal pain and weakness. In the ED, vitals were notable for a blood pressure of 139/90 mmHg, tachycardia with a heart rate of 120 bpm, respiratory rate of 18 bpm, and oxygen saturation of 98% on room air; the patient was febrile with a temperature of 39.2 degrees Celsius. The physical exam was notable for normal neck range of motion, tachycardic heart rate with a regular rhythm, clear lung sounds with adequate air entry with an absence of wheezing, mild abdominal tenderness to the epigastric area and periumbilical fields, and normal mentation and neurologic function. Laboratory studies were remarkable for mild leukocytosis with a WBC of 12.7 K/mm^3^ (reference range 4.5 - 11), with unremarkable electrolytes and adequate kidney function. Urinalysis was significant for cloudy urine with moderate blood, large leukocyte esterase, with >100 white blood cells, and occasional bacteria, and the patient received empiric antibiotic therapy with intravenous ceftriaxone and metronidazole for urinary tract infection. EKG was significant for sinus tachycardia at 123 beats per minute without evidence of acute ischemia. The patient received intravenous fluids and received acetaminophen for pain. The patient underwent computed tomography (CT) of the abdomen and pelvis with intravenous contrast, which was significant for questionable bilateral perinephric fat stranding.

The patient was accepted by the Medicine team for admission for pyelonephritis; however, during her time in the ED following the completion of the CT scan, she began to develop respiratory distress. The patient appeared anxious, was tachypneic, and hypoxic on room air. The patient was found to have diffuse urticaria. She was placed on supplemental oxygen with a nasal cannula and received diphenhydramine, prednisone, and epinephrine; however, despite these treatments, the patient developed worsening respiratory distress and her lips appeared cyanotic. Due to hypoxic respiratory failure, the decision was made to intubate. Unfortunately, following intubation with etomidate and rocuronium, the patient was found to have pulseless ventricular tachycardia with a code blue initiated. She received a dose of epinephrine and amiodarone during CPR, requiring three cardioversions, which later demonstrated atrial fibrillation with a rapid ventricular response. Pulses were palpated, and the patient was ultimately admitted to the medical intensive care unit (MICU) for further workup and management of suspected anaphylactic shock.

The patient was treated in the MICU for three days with the assistance of Cardiology. She was initiated on a heparin drip and aspirin with close monitoring of troponin levels. The patient eventually returned to normal sinus rhythm on the cardiac monitor, and a 2D echocardiogram demonstrated a left ventricular ejection fraction of 55-60%. Due to clinical stability, Cardiology recommended no intervention, with cessation of heparin drip. The patient was extubated successfully and was transferred to the medical floor.

However, following the transfer, a rapid response was called for dyspnea. On evaluation, the patient was tachycardic at 110 bpm, with diffuse expiratory wheezing appreciated on physical exam. Given her clinical picture, she was suspected to have a second event of anaphylaxis and received high-dose steroids, diphenhydramine, ipratropium/albuterol nebulizers, and racemic epinephrine. A bedside ultrasound was performed, which was significant for B-lines, and the patient received intravenous furosemide. Given the high risk for re-intubation, Critical Care was consulted and decided to transfer the patient back to the MICU for closer monitoring.

**Figure 1:**
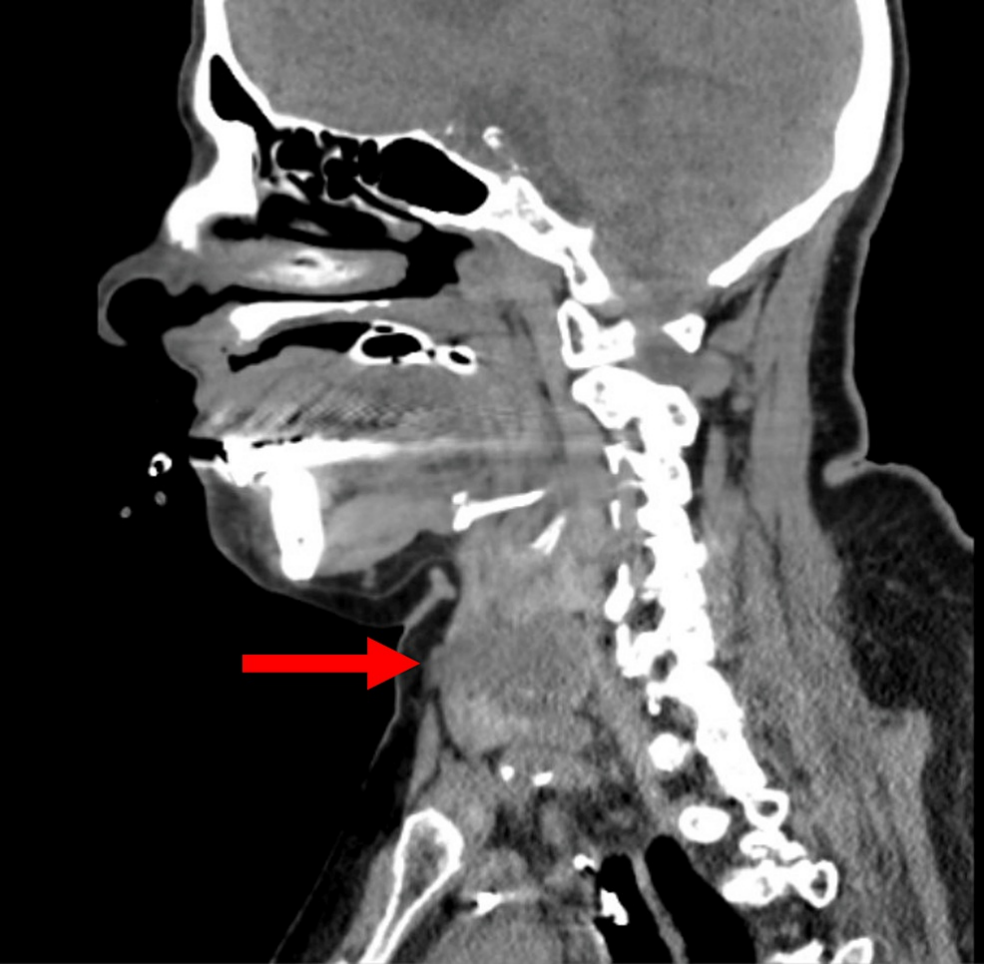
Computed tomography neck and soft tissue without contrast: notable for a heterogeneous thyroid with a focal nodule in the right thyroid lobe measuring up to 3 centimeters (red arrow)

**Figure 2:**
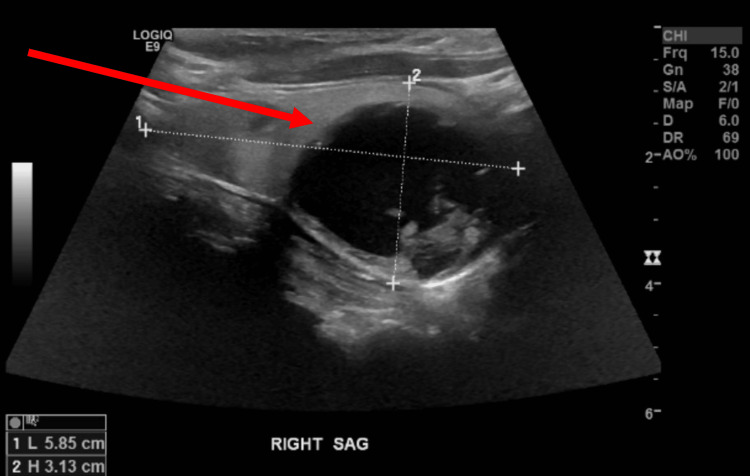
Thyroid ultrasound notable for a large dominant complex solid and cystic mass in the right thyroid gland (red arrow)

Given the patient’s recurrent anaphylaxis-like symptoms, additional differentials were considered, such as chronic obstructive pulmonary disease (COPD), given the patient’s extensive tobacco use history. She received steroids, ipratropium/albuterol nebulizers, cetirizine, and montelukast as empiric treatment. Thyroid studies were completed, which were notable for thyroid-stimulating hormone (TSH) of 0.019 mcInltUnit/mL (reference range 0.450 - 5.330), free T4 of 1.63 ng/dL (reference range 0.61 - 1.12), T3 of 3.25 pg/mL (reference range 2.30 - 4.20), and a negative thyroid stimulating immunoglobulin (TSI) of <0.10 IntlUnit/L (reference range 0.00 - 0.55). Urinary 5-hydroxy indoleacetic acid (5-HIAA) sampling was obtained to evaluate for carcinoid syndrome, which was unremarkable with a value of 5.9 mg/24 hr (reference range 0.0 - 14.9). Allergen testing was unremarkable. A CT neck without contrast demonstrated a 3.3 x 2.6cm complex R thyroid nodule (Figure 1). Ultrasound of the thyroid was significant for a large dominant complex solid and cystic mass in the right thyroid gland (Figure 2). Subsequently, the Endocrinology team was consulted for a thyroid nodule, and there was consideration for euthyroid-sick syndrome versus thyrotoxicosis with toxic adenoma activated by iodinated contrast and/or amiodarone. Another consideration for the thyroid nodule was lung cancer metastasis given her history of malignancy, although this was less likely. A radioiodine uptake scan was avoided as the patient received contrast imaging on admission. Per the patient’s son, he was unaware of thyroid issues or allergic reactions in the past and denied a history of his mother experiencing weight loss, palpitations, anxiety, or diarrhea. The patient received methimazole 5 mg oral daily and plans were made for outpatient follow-up and repeat thyroid function testing upon discharge.

## Discussion

Iodine represents a rate-limiting trace element for the synthesis of iodine-containing thyroid hormones, triiodothyronine (T3) and thyroxine (T4), and is mainly cleared via the kidneys and thyroid gland [4]. When in excess, iodine inhibits thyroid hormone secretion via inhibition of organification, with in vitro studies demonstrating iodide-driven inhibition of both the calcium phosphatidylinositol 4,5-bisphosphate cascade and the cyclic adenosine monophosphate cascade, as well as downregulation of the sodium/iodide transporter [5]. This is considered the physiologic reaction when iodine is in excess amounts, which is referred to as the "Wolff-Chaikoff Effect." Alternatively, individuals may develop hyperthyroidism when administered iodine, via amiodarone or intravenous contrast for imaging, and when this occurs this is considered the "JB phenomenon" [2]. This mechanism is considered to occur due to portions or nodules of the thyroid gland acting in an autonomous fashion and subsequently leading to an increase in the production of thyroid hormones [6].

Considering an excess of iodine is an instrumental part of the pathophysiology of the JB phenomenon, chronic kidney disease, and end-stage renal disease are also considered predisposing conditions as iodine is renally excreted [2]. Lastly, exposure to products that contain high levels of iodine such as iodinated contrast media or iodinated antiseptic solutions increases the risk of developing the JB phenomenon [1].

It is of utmost importance to consider underlying thyroid diseases like multinodular goiter, Graves’ disease, use of iodine-rich food items, and medications like amiodarone and Lugol's iodine to be considered while evaluating the JB phenomenon [2]. The most common underlying thyroid abnormality in patients with the JB phenomenon is multinodular goiter and the least common is Graves’ disease [2]. A thorough review of the patient’s symptoms via a detailed history and physical is key to joining the dots in establishing a correlation between symptoms and occurrence, and ultimately preventing the JB phenomenon. Our patient had normal thyroid-stimulating immunoglobulin (TSI) of <0.10 IntlUnit/L (reference range 0.00 - 0.55) and no clinical evidence of Graves' disease at the time of presentation, hence ruling out Graves' disease as a cause. Our patient had symptoms of a urinary tract infection for which she was receiving antibiotics, and this could have been a questionable initial stressor. However, she demonstrated no signs of viral illness, or thyroid gland tenderness on examination, making viral thyroiditis an unlikely cause. Our patient had no previous radioactive scans, or history of use of chronic amiodarone, lithium, interferon-α, and interleukin-2, which commonly causes painless thyroiditis. With thyroid malignancy rarely causing hyperfunctioning thyroid and the absence of any thyroid mass, absence of weight loss, or dysphagia in our patient, malignancy was a less likely differential [7]. As a female with advanced age and with the initial stressor of urinary tract infection, subsequently followed by iodinated contrast use of CT imaging of the neck, this may have precipitated the occurrence of the JB phenomenon. Evaluating risk factors with thorough history aids in establishing a correlation between such events.

The JB phenomenon is primarily self-limited in most cases with the mainstay of management being source control and discontinuation of iodine exposure [8]. Additionally, preventative management is key such as utilizing non-contrast CT or MRI imaging when possible. Current trials have not mounted enough evidence on the use of thioamide or percolate for the treatment of the JB phenomenon, thereby frequent lab monitoring of TSH and ft4 post-iodinated contrast use can be helpful [8]. If medication-induced, such as an instance of amiodarone use, management must be individualized based on the severity of cardiac history. Sudden withdrawal can potentiate more harm by worsening hyperthyroid signs and symptoms. In such cases, after a euthyroid state is achieved, radioactive iodine or total thyroidectomy can be considered [8]. Another modality available is plasmapheresis, which has shown beneficial effects for the removal of excess hormones in many cases [8].

## Conclusions

In summary, the JB phenomenon is a rare event most commonly observed in patients exposed to iodinated contrast media, specifically those with an underlying thyroid condition. The resulting thyrotoxicosis can be dangerous to the patient, and therefore it is critical to quickly identify and treat hyperthyroidism. It is important to be wary of this event when administering iodinated contrast media and other iodide-containing products to patients with a known history of thyroid disease and those with no known history of thyroid disease since the latter population is also susceptible, as seen with our patient.
